# Outcomes and Predictive Factors Associated with Adequacy of Antimicrobial Therapy in Patients with Central Line-Associated Bloodstream Infection

**DOI:** 10.3389/fpubh.2016.00284

**Published:** 2016-12-23

**Authors:** Paula Kiyomi Onaga Yokota, Alexandre Rodrigues Marra, Talita Rantin Belucci, Elivane da Silva Victor, Oscar Fernando Pavão dos Santos, Michael B. Edmond

**Affiliations:** ^1^Division of Medical Practice, Hospital Israelita Albert Einstein, São Paulo, Brazil; ^2^Instituto Israelita de Ensino e Pesquisa Albert Einstein, Hospital Israelita Albert Einstein, São Paulo, Brazil; ^3^Department of Internal Medicine, University of Iowa Carver College of Medicine, Iowa City, IA, USA

**Keywords:** central line-associated infection, inadequate antimicrobial therapy, mortality, blood stream infection, central venous catheters

## Abstract

**Background:**

Central venous catheters are significant risk factors for bloodstream infection (BSI), which are directly associated with increased morbidity and mortality.

**Methods:**

This study was a retrospective cohort study for the time period of July 2011–June 2014 in patients with central line-associated bloodstream infection (CLABSI) to determine the microbiological profile and antimicrobial adequacy of patients with CLABSI in a tertiary hospital.

**Results:**

One hundred and twenty-one CLABSI cases were identified. Ninety-two percent (*n* = 111) of patients had monomicrobial BSI. Gram-negative bacteria were the most prevalent (49%, *n* = 63), with *Klebsiella* spp. predominating (30%, *n* = 19). Among the Gram-positive bacteria (*n* = 43, 33%), coagulase-negative staphylococci was the major pathogen (58%, *n* = 25), and all isolates were methicillin resistant. Antimicrobial therapy was assessed as adequate in 81% (*n* = 98) of cases. In-hospital mortality was 36% (*n* = 43 cases).

**Conclusion:**

Our CLABSI patients had a high mortality, although antimicrobial therapy was appropriate. Gram-negative bacteria were responsible for almost half of the cases and there was a high rate of bacteria resistance to extended-spectrum antibiotics.

## Introduction

Central venous catheters (CVCs) are essential for the care of seriously ill patients (1, 2). However, CVCs are significant risk factors for bloodstream infection (BSI), which are directly associated with increased morbidity and mortality, length of hospital stay, and medical and hospital costs (2–4). Each year in the United States, there are an estimated 80,000 new cases of central line-associated bloodstream infection (CLABSI), resulting in over 28,000 deaths among patients in intensive care units, with an attributable cost of more than 2.3 billion dollars (5).

To improve antimicrobial therapy for BSI, it is necessary to understand the microorganism profile as well as the antimicrobial susceptibility of these microorganisms (6–8). In a recent publication from our hospital, which analyzed the impact of the implementation of a sepsis protocol, 60% of our patients developed severe sepsis and septic shock outside of the Emergency Department, which increases the probability of nosocomial infection with antibiotic-resistant microorganisms (9, 10). There are serious concerns about the rapid increase of multidrug resistant bacteria, mainly the Gram-negative microorganisms (10).

Thus, the objectives of this study were to determine the microbiological profile and antimicrobial adequacy of patients with CLABSI in a tertiary hospital.

## Materials and Methods

This study was conducted in a 629-bed tertiary care (Hospital Israelita Albert Einstein), private hospital in São Paulo, Brazil, with approximately 194,000 patient-days yearly. This study was approved by the Institutional Review Board (CAAE # 40993115.8.0000.0071) with a waiver of informed consent.

We performed a retrospective cohort study for the time period of July 2011–June 2014, in patients with central line-associated bloodstream infection.

### Definitions

Central lines were defined using the definition of the National Healthcare Safety Network (NHSN) (11). The NHSN methodology for CLABSI employs active surveillance with standardized definitions by infection preventionists. The definition of CLABSI was an NHSN defined laboratory-confirmed bloodstream infection that was not secondary to an infection at another body site (11).

### The ESKAPE Group

ESKAPE group pathogens were defined as the following: vancomycin resistant *Enterococcus faecium*, methicillin-resistant *Staphylococcus aureus*, extended-spectrum beta-lactamase—producing *Escherichia coli* and *Klebsiella* species, *Klebsiella pneumoniae* carbapenemase-hydrolyzing beta-lactamases, multidrug resistant *Acinetobacter* species, resistant *Pseudomonas aeruginosa*, and *Enterobacter species* (12).

### Antimicrobial Therapy

As in our previous study (10), antimicrobial therapy was considered appropriate if the bacterial species identified in blood culture was susceptible to at least one of the antibiotics administered within 24 h after the collection of culture. If the isolated microorganism was not susceptible by *in vitro* testing to the antibiotic(s) used, the therapy was considered inadequate. The microbiology laboratory has an alert system to notify physicians of patients with positive blood culture and their Gram stain results. Also the proper antimicrobial dose, proper interval administration, monitoring of drug levels when appropriate, and avoidance of unwanted drug interactions were evaluated (6) by the pharmaceutical hospital in our medical routine.

### Microbiological Methods

All samples were identified by manual or automated methods and confirmed using the Vitek 2 system (bioMerieux Vitek, Inc., Hazelwook, MO, USA). The same organism with identical antimicrobial susceptibility profiles from the same or different anatomic sites in the same patient was considered a single isolate. Antimicrobial susceptibility testing was performed by an automated method or by disk diffusion as described by the Clinical and Laboratory Standards Institute (13).

### Statistical Analysis

Continuous variables were described by mean and SD or median and interquartile range (IQR). Categorical variables were described as absolute numbers and percentages. Logistic regression models were used to investigate variables associated with death or adequate antimicrobial therapy. Variables with *P* < 0.1 in the univariate analysis were included in the multivariate models, which were submitted to stepwise method to select the best subset of predictors to each outcome. Results were presented as odds ratios and 95% confidence intervals. The level of significance was set at 0.05 and analyses were performed with statistical package R (14).

## Results

A total of 121 CLABSI cases were identified from July 2011 to June 2014. The mean age of the cases was 67 years (SD = 18), and the median length of hospital stay was 43 days (exact median cannot be calculated as two patients remain hospitalized). Of the cases, 56% (*n* = 68) were male. ESKAPE group pathogens accounted for 21% (*n* = 25) of the cases. Polymicrobial CLABSIs accounted for 9% (*n* = 11) of the cases. Antimicrobial therapy was assessed as adequate in 81% (*n* = 98) of cases. Cardiac dysfunction occurred in 63% (*n* = 76) and respiratory dysfunction occurred in 52% (*n* = 63). In-hospital mortality was 36% (*n* = 43 cases) (Table 1).

**Table 1:** **Demographic and clinical characteristic of patients with central line-associated bloodstream infection**.

Variables	(*n* = 121)	
	*n*	%
Age—mean (SD)	67	18
Length of stay—median (IQR)	43	22–81^a^
Male	68	56.2
ESKAPE group pathogen	25	20.7
Polymicrobial infection	11	9.1
Appropriate antibiotic therapy	98	81.0
Respiratory dysfunction	63	52.1
Cardiac dysfunction	76	62.8
Renal dysfunction	38	31.4
Liver dysfunction	36	29.8
Hematologic dysfunction	37	30.6
Crude mortality rate	43	35.5

*ESKAPE, Enterococcus, Staphylococcus aureus, Klebsiella pneumoniae, Pseudomonas aeruginosa, Enterobacter spp.; IQR, interquartile range*.

*^a^Minimum and maximum length of stay—2 and 600 days*.

In univariate analysis, variables potentially associated with death (*P* < 0.1) among patients with CLABSI (Table 2) were age, liver dysfunction, cardiac dysfunction, renal dysfunction, hematologic dysfunction, respiratory dysfunction, heart failure, polymicrobial infection, and adequate antimicrobial infection. In the final multivariate model, after stepwise selection, the following variables were statistically significant: age (OR: 1.1, *p* < 0.01), liver dysfunction (OR: 4.3, *p* = 0.01); hematologic dysfunction (OR: 6.1, *p* = 0.01); and renal dysfunction (OR: 10.8, *p* < 0.01); renal failure (OR: 0.2, *p* = 0.03); cancer (OR: 4.3, *p* = 0.04); and polymicrobial infection (OR: 5.9, *p* = 0.04).

**Table 2:** **Risk factors associated with death in patients with central line-associated bloodstream infection**.

Variables	Survival (*n* = 78) *n* (%)	Death (*n* = 43) *n* (%)	Univariate analysis			Multivariate analysis		
			OR	CI 95% (LL–UL)	*P*	OR	CI 95% (LL–UL)	*P*
Age (years), mean (SD)	64.7 (19.4)	71.2 (14.4)	1.0	(1.0–1.04)	0.06	1.1	(1.0–1.1)	<0.01
Male	41 (52.6)	27 (62.8)	1.5	(0.7–3.3)	0.28			
Organs dysfunction								
Liver dysfunction	16 (20.5)	20 (46.5)	3.4	(1.5–7.6)	0.01	4.3	(1.3–16.0)	0.01
Cardiac dysfunction	41 (52.6)	35 (81.4)	4.0	(1.6–9.6)	0.01	3.1	(0.7–15.2)	0.16
Renal dysfunction	14 (17.9)	24 (55.8)	5.8	(2.5–13.3)	<0.01	10.8	(2.5–60.0)	<0.01
Hematologic dysfunction	19 (24.4)	18 (41.9)	2.2	(1.0–5.0)	0.05	6.1	(1.6–27.1)	0.01
Respiratory dysfunction	34 (43.6)	29 (67.4)	2.7	(1.2–5.8)	0.01			
Comorbidities								
Diabetes mellitus	27 (34.6)	14 (32.6)	0.9	(0.4–2.0)	0.82	0.3	(0.1–1.0)	0.057
Renal failure	15 (19.2)	6 (14.0)	0.7	(0.2–1.9)	0.47	0.2	(0.1–0.8)	0.03
Hypertension	33 (42.3)	13 (30.2)	0.6	(0.3–1.3)	0.19			
Heart failure	6 (7.7)	10 (23.3)	3.7	(1.2–10.9)	0.02	18.3	(2.8–182.0)	0.01
Cancer	27 (34.6)	19 (44.2)	1.5	(0.7–3.2)	0.30	4.3	(1.2–19.7)	0.04
Transplantation	11 (14.1)	2 (4.7)	0.3	(0.1–1.4)	0.13			
Polymicrobial infection	4 (5.1)	7 (16.3)	3.6	(1.1–13.1)	0.05	5.9	(1.1–34.8)	0.04
ESKAPE^a^ pathogen causing bloodstream infection	19 (24.4)	6 (14.0)	0.5	(0.2–1.4)	0.18			
Adequate antimicrobial therapy	67 (85.9)	31 (72.1)	0.4	(0.2–1.1)	0.07			

*^a^ESKAPE, Enterococcus, Staphylococcus aureus, Klebsiella pneumoniae, Pseudomonas aeruginosa, Enterobacter spp.; OR, odds ratio; CI, confidence interval; LL, lower limit; UL, upper limit*.

Appropriate antimicrobial treatment was administered in 81% of patients (*n* = 98). All lines in patients with confirmed CLABSI were removed. No variable predicted antimicrobial inadequacy (Table 3). Although there was a trend toward increased mortality with inadequate antimicrobial therapy, it was not statistically significantly different.

**Table 3:** **Risk factors associated with inadequate antimicrobial therapy in patients with central line-associated bloodstream infection**.

Variables	Adequate antimicrobial therapy		Univariate analysis			Multivariate analysis		
	No (*n* = 23) *n* (%)	Yes (*n* = 98) *n* (%)	OR	CI 95% (LL–UL)	*P*	OR	CI 95% (LL–UL)	*P*
Male	15 (65.2)	53 (54.1)	0.6	(0.2–1.6)	0.34			
Age (years), mean (SD)	70.7 (17.5)	66.2 (18.1)	0.99	(0.96–1.1)	0.29			
**Organs dysfunctions**								
Liver dysfunction	8 (34.8)	28 (28.6)	0.8	(0.3–2.0)	0.56			
Cardiac dysfunction	16 (69.6)	60 (61.2)	0.7	(0.3–1.8)	0.46			
Renal dysfunction	9 (39.1)	29 (29.6)	0.7	(0.3–1.7)	0.38			
Hematologic dysfunction	7 (30.4)	30 (30.6)	1.0	(0.4–2.7)	0.99			
Respiratory dysfunction	12 (52.2)	51 (52.0)	1.0	(0.4–2.5)	0.99			
Polymicrobial infection	1 (4.3)	10 (10.2)	2.5	(0.3–20.6)	0.39			
ESKAPE pathogen causing bloodstream infection	2 (8.7)	23 (23.5)	3.2	(0.7–14.8)	0.13	3.2	(0.7–14.8)	0.13

*ESKAPE, Enterococcus, Staphylococcus aureus, Klebsiella pneumoniae, Pseudomonas aeruginosa, Enterobacter spp.; OR, odds ratio; CI, confidence interval; LL: lower limit; UL: upper limit*.

Among patients identified with CLABSI, 92% (*n* = 111) of patients had monomicrobial BSI. Gram-negative bacteria were the most prevalent (49%, *n* = 63), with *Klebsiella* spp. predominating (30%, *n* = 19), followed by *P. aeruginosa* (17.5%, *n* = 11), *E. coli* (15.9%, *n* = 10), and *Enterobacter* spp. (12.7%, *n* = 8). Among the Gram-positive bacteria (*n* = 43, 33%), coagulase-negative staphylococci was the major pathogen (58%, *n* = 25), and all isolates were methicillin resistant, followed by *S. aureus* (23.3%, *n* = 10) and *Enterococcus* spp. (14.0%, *n* = 6). The great majority of *S. aureus* and *Enterococcus* spp. were methicillin resistant (90%, *n* = 9) and vancomycin resistant (83.3%, *n* = 5), respectively. Among the fungi (18%, *n* = 23), *Candida parapsilosis* and *Candida albicans* were the most prevalent (30%, *n* = 7, and 22%, *n* = 5, respectively).

## Discussion

Our findings identified the pathogen profile in patients with CLABSI. Regardless of the hospital unit where CLABSI occurred, Gram-negative bacteria, were the most common pathogens, especially *Klebsiella* spp. (30% of all Gram-negative pathogens), and nearly all of which (95%) were resistant to the third- and fourth-generation cephalosporins. This differs from international studies, including a meta-analysis that assessed 18 studies carried out in different intensive care units, which showed that Gram-positive bacteria are the most prevalent pathogens (15). In the Brazilian Surveillance and Control of Pathogens of Epidemiological Importance Project, the spectrum of pathogens was similar to the present study (16). Fisman et al. demonstrated that geography has a direct influence on the development of BSI caused by Gram-negative bacteria (17).

In our earlier investigation, evaluating patients with severe sepsis and septic shock, the bacterial profile was similar, with a predominance of Gram-negative organisms; however, there was a greater prevalence of *E. coli* (10). There was a great difference in the proportion of BSI caused by fungi, only 1.5% of cases in the previous study (10), compared to 18% in the current study with 65% in-hospital mortality.

The most common microorganisms responsible for CLABSI change over time. For example, the best practices in ICU care have reduced the proportion of CLABSI due to Gram-positive bacteria (15, 18, 19).

The higher prevalence of *Klebsiella* spp. was also identified in a previous study (16). Also previous findings described by Correa et al. warned off the increased resistance of *K. pneumoniae* (20) and reinforced by the European Antimicrobial Resistance Surveillance System in its latest report of hospitals (2011–2012), which was analyzed in 29 European countries (21).

Heart failure was shown to be an independent risk factor for death. In addition, the presence of liver, renal, and hematological dysfunctions significantly increased the risk of death. Moreover, concomitant toxicity of antimicrobial treatment of multidrug resistant microorganisms, particularly with prolonged duration of therapy, adds to the morbidity of the systemic inflammatory response induced by the pathogen (6, 18).

Antimicrobial therapy in a previous study was an independent predictor for lower mortality in patients with severe sepsis and septic shock (10). In our study, inadequacy of therapy showed a trend toward increased mortality. However, the characteristics of these patients show us that appropriate initial antimicrobial treatment of these cases portend a greater chance of error, when polymicrobial infection or ESKAPE pathogens are possible.

The limitations of this study include performance in a single center, which may not allow generalization to other hospitals. Although some information was retrospectively obtained from medical records, the patients were followed prospectively by the hospital epidemiology unit.

In conclusion, our CLABSI patients had a high mortality, although antimicrobial therapy was appropriate. Gram-negative bacteria were responsible for almost half of the cases and there was a high rate of bacteria resistance to extended-spectrum antibiotics.

## Ethics Statement

This study was approved by the Institutional Review Board of Albert Einstein Institute of Research (CAAE # 40993115.8.0000.0071) with a waiver of informed consent.

## Author Contributions

PY, AM, TB, and EV participated in the data collected and data analysis. PY, AM, OS, and ME participated in the design and coordination. PY, AM, EV, and ME helped to draft the manuscript and to provide critical review of the manuscript. All authors read and approved the final manuscript.

## Conflict of Interest Statement

The authors declare that the research was conducted in the absence of any commercial or financial relationships that could be construed as a potential conflict of interest.

## References

[1] Blot K Bergs J Vogelaers D Blot S Vandijck D. Prevention of central line-associated bloodstream infections through quality improvement interventions: a systematic review and meta-analysis. Clin Infect Dis (2014) 59:95–105. 10.1093/cid/ciu239 24723276 PMC4305144

[2] Hejjej Z Nasri M Sellami W Gharsallah H Labben I Ferjani M. Incidence, risk factors and microbiology of central vascular catheter-related bloodstream infection in an intensive care unit. J Infect Chemother (2014) 20:163–8. 10.1016/j.jiac.2013.08.001 24508422

[3] Marra AR Cal RGR Durão MS Correa L Guastelli LR Moura DF Jr Impact of a program to prevent central line-associated bloodstream infection in the zero tolerance era. Am J Infect Control (2010) 38:434–9. 10.1016/j.ajic.2009.11.012 20226570

[4] Burden AR Torjman MC Dy GE Jaffe JD Litmann JJ Nawar F Prevention of central venous catheter-related bloodstream infection: is it time to add simulation training to the prevention bundle? J Clin Anesth (2012) 24:555–60. 10.1016/j.jclinane.2012.04.006 23101770

[5] Pronovost P Needham D Berenholtz S Sinopoli D Chu H Cosgrove S An intervention to decrease catheter-related bloodstream infection in the ICU. N Engl J Med (2006) 355(26):2725–32. 10.1056/NEJMoa061115 17192537

[6] Ibrahim EH Sherman G Ward S Fraser VJ Kollef M. The influence of inadequate antimicrobial treatment of bloodstream infections on patient outcomes in the ICU setting. Chest (2000) 118:146–55. 10.1378/chest.118.1.146 10893372

[7] Zaragoza R Artero A Camarena JJ Sancho S González R Nogueira JM The influence of inadequate empirical antimicrobial treatment on patients with bloodstream infection in an intensive care unit. Clin Microbiol Infect (2003) 9:412–8. 10.1046/j.1469-0691.2003.00656.x 12848754

[8] Kang CI Kim SH Kim HB Park SW Choe YJ Oh MD *Pseudomonas aeruginosa* bacteremia: risk factors for mortality and influence of delayed receipt of effective antimicrobial therapy on clinical outcome. Clin Infect Dis (2003) 37:745–51. 10.1086/377200 12955633

[9] Shiramizo SC Marra AR Durao MS Paes AT Edmond MB Pavao dos Santos OF Decreasing mortality in severe sepsis and septic shock patients by implementing a sepsis bundle in a hospital setting. PLoS One (2011) 6:e26790 10.1371/journal.pone.0026790 22073193 PMC3207817

[10] Yokota PKO Marra AR Martino MDV Victor ES Durao MD Edmond MB Impact of appropriate antimicrobial therapy for patients with severe sepsis and septic shock – a quality improvement study. PLoS One (2014) 9(11):e104475 10.1371/journal.pone.0104475 25375775 PMC4222820

[11] Centers for Disease Control and Prevention. Device-Associated Module. Bloodstream Infection Event (Central Line-Associated Bloodstream Infection and Non-Central Line Associated Bloodstream Infection). Atlanta, GA: CDC (2015). Available from: http://www.cdc.gov/nhsn/PDFs/pscManual/4PSC_CLABScurrent.pdf

[12] Boucher HW Talbot GH Bradley JS Edwards JE Gilbert D Rice LB Bad bugs, no drugs: no ESKAPE! An update from the Infectious Disease Society of America. Clin Infect Dis (2009) 48:1–12. 10.1086/595011 19035777

[13] Clinical and Laboratory Standards Institute. Performance Standards for Antimicrobial Susceptibility Testing: Sixteenth Informational Supplement. CLSI Document M100-S16. Wayne, PA: CLSI (2006).

[14] R Core Team. R: A Language and Environment for Statistical Computing. Vienna, Austria: R Foundation for Statistical Computing (2015). Available from: http://www.R-project.org/

[15] Ziegler MJ Pellegrini DC Safdar N. Attributable mortality of central line associated bloodstream infection: systematic review and meta-analysis. Infection (2015) 43:29–36. 10.1007/s15010-014-0689-y 25331552

[16] Marra AR Camargo Aranha LF Pignatari ACC Sukiennik T Behar PR Medeiros EA Nosocomial bloodstream infection in Brazilian hospitals: analysis of 2,563 cases from a prospective Nationwide surveillance study. J Clin Microbiol (2011) 49:1866–71. 10.1128/JCM.00376-11 21411591 PMC3122653

[17] Fisman D Patrozou E Cameli Y Perencevich E Tuite AR Mermel LA Geographical variability in the likelihood of bloodstream infections due to Gram-negative bacteria: correlation with proximity to the equator and health care expenditure. PLoS One (2014) 9(12):e114548. 10.1371/journal.pone.0114548 25521300 PMC4270641

[18] Anderson DJ Moehring RW Sloane R Schmader KE Weber DJ Fowler VG Jr Bloodstream infections in community hospitals in the 21^st^ century: a multicenter cohort study. PLoS One (2014) 9(3):e91713. 10.1371/journal.pone.0091713 24643200 PMC3958391

[19] Climo MW Yokoe DS Warren DK Perl TM Bolon M Herwaldt LA Effect of daily chlorhexidine bathing on hospital-acquired infection. N Engl J Med (2013) 368:533–42. 10.1056/NEJMoa1113849 23388005 PMC5703051

[20] Correa L Martino MD Siqueira I Pasternak J Gales AC Silva CV A hospital-based matched case-control study to identify clinical outcome and risk factors associated with carbapenem-resistant *Klebsiella pneumoniae* infection. BMC Infect Dis (2013) 13:80. 10.1186/1471-2334-13-80 23398691 PMC3574843

[21] European Centre for Disease Prevention and Control. ECDC Surveillance Report. Point Prevalence Survey of Healthcare-Associated Infections and Antimicrobial Use in European Acute Care Hospitals 2011-2012. (2015). Available from: http://ecdc.europa.eu/en/publications/Publications/healthcare-associated-infections-antimicrobial-use-PPS.pdf 10.2807/ese.17.46.20316-en 23171822

